# Knowledge and Observed Practices of Hospital‐Acquired Infection Prevention Guidelines Among Nurses Working in Neonatal Intensive Care Units in Jordan: A Cross‐Sectional Observational Study

**DOI:** 10.1155/tswj/8882300

**Published:** 2026-03-18

**Authors:** Abedallah Y. Kasem, Huda Kh. Ahmad, Nadin M. Abdel Razeq, Alaa O. Oteir, Eihab A. Khasawneh, Wafaa Y. AlGhazawi

**Affiliations:** ^1^ Department of Maternal and Child Health Nursing, Faculty of Nursing, Jordan University of Science and Technology, Irbid, Jordan, just.edu.jo; ^2^ Nusayba Al-Mazinia College for Nursing and Midwifery, Ministry of Health, Irbid, Jordan, moh.gov.jo; ^3^ Department of Maternal and Child Health Nursing, School of Nursing, The University of Jordan, Amman, 11942, Jordan, ju.edu.jo; ^4^ Department of Allied Medical Sciences, Faculty of Applied Medical Sciences, Jordan University of Science and Technology, Irbid, Jordan, just.edu.jo

**Keywords:** hospital-acquired infection, infection prevention guidelines, knowledge, neonates, nurses, practice

## Abstract

**Background:**

Hospital‐acquired infection (HAI) is one of the leading causes of mortality and morbidity in neonates. Knowledge and practice of infection prevention guidelines are the cornerstone to minimizing HAI among sick and preterm neonates in intensive care units (NICU).

**Objectives:**

To assess neonatal nurses’ knowledge and observed practices of HAI prevention guidelines and to determine their most important contributing factors.

**Design:**

A cross‐sectional descriptive observational design was applied.

**Setting:**

NICUs of two large teaching hospitals in the northern region of Jordan.

**Participants:**

Neonatal nurses (*n* = 85) working full‐time at the selected NICUs.

**Primary and Secondary Outcome Measures:**

Neonatal nurses’ knowledge level and observed practices regarding HAI prevention guidelines were assessed through a self‐administered questionnaire and a structured observation checklist developed in accordance with the WHO and CDC infection control guidelines.

**Results:**

Knowledge levels about HAI prevention guidelines were rated good in 65% of participants, fair in 34%, and poor in 1%. Observed HAI prevention practices were good in 12.9% of participating nurses, fair in 71.8%, and poor in 15.3% of participants. No significant correlation was found between nurses’ knowledge and their observed practice. However, knowledge was significantly correlated with workplace and attending infection prevention courses (*p* < 0.05), while good practice levels were positively correlated with educational level (*p* = 0.044), but negatively correlated with age, years of experience, and workplace (*p* = 0.013, *p* = 0.023, *p* = 0.001, respectively).

**Conclusion:**

Despite being knowledgeable, NICU nurses in our study demonstrated suboptimal practices of HAI prevention guidelines. This knowledge–practice gap highlights the importance of reinforcing infection prevention through ongoing education, supportive institutional measures, and regular monitoring. Such efforts may help strengthen adherence to safe infection prevention practices and enhance the quality of neonatal care. Future studies with larger and more diverse samples are recommended to validate these findings and explore contextual factors influencing practice.

## 1. Introduction

Hospital‐acquired infection (HAI), also known as nosocomial infection (NI) and health care‐associated infection (HCAI), is defined as “*an infection occurring in a patient during the process of care within a health care facility that was not present or incubating at the time of admission*” [1]. HAIs are a global health concern, contributing to substantial morbidity and mortality among all age groups, increasing the burden and utilization loads on healthcare systems, prolonging hospital stays, and magnifying treatment and service costs offered for or by the affected patients [2–5].

A recent meta‐analysis of 400 articles published between 2000 and 2021 estimated the prevalence of HAIs globally to be 0.14, with an annual increase of 0.06% [6]. In neonates and NICUs, the point estimate of global HAI prevalence rate was reported as 0.69 and 0.44, respectively [6]. Prevalence rates, however, varied considerably across countries and the literature, with the highest rates documented in the low‐ and middle‐income countries, specifically those in the Eastern Mediterranean and African Regions [6]. In some reports, the incidence of HAIs in NICUs has been noted to reach up to 47% [7, 8].

Surveillance data from large neonatal networks highlight that reduction in HAI rates and improvements in related neonatal outcomes are primarily achieved through preventive rather than curative approaches. The most effective initiatives emphasize healthcare workers’ adherence to evidence‐based practices [9]. Engagement of trained professionals on infection control guidelines, nurses in particular, is central to successfully implementing HAI prevention guidelines in every acute care facility [10].

As frontline providers in NICUs, nurses play a key role in maintaining neonatal safety through consistent adherence to simple HAI prevention practices, such as hand hygiene (HH) and the use of personal protective equipment (PPE), which can significantly reduce HAI incidence among neonates [9]. Grounded in Florence Nightingale’s environmental theory, nurses who possess knowledge of contagion processes and environmental cleanliness are able to translate this knowledge into effective practices that foster a clean, health care environment and support optimal recovery while preventing infection, which is nowadays known as infection control guidelines [11].

Jordan is a lower‐middle‐income country according to the World Bank classification of 2022 [12]. Jordan’s reports about neonatal mortality indicate that the majority of neonatal deaths occur in the NICU and that sepsis is one important factor contributing to neonatal mortality [13]. In Jordan, it is less clear how much of the sepsis rate is contributed to by HAI among the neonates in NICU. However, it is well‐known that neonatal sepsis accounts for 16% [13] to 27% [14] of neonatal mortality causes in NICUs.

Yet, a detailed description of the HAI prevention practices by the healthcare workers in the NICU in Jordan is surprisingly unavailable; without such information, quality improvement interventions targeting HAI prevention would be challenging. Therefore, this study was conducted as a response to the limited research‐based information on the NICU nurses’ knowledge and actual practices of infection prevention guidelines. It aims to assess neonatal nurses’ knowledge and observed practices of HAI prevention guidelines and to determine their most important contributing factors.

## 2. Methods

### 2.1. Design

This was a cross‐sectional descriptive observational study.

### 2.2. Setting

The study was conducted in the NICUs of two tertiary teaching hospitals in Irbid, northern Jordan. Irbid is the second‐largest city in Jordan with a population of around two million [15]. The first hospital (Hospital 1) is a university‐affiliated hospital, and the second (Hospital 2) is a governmental teaching hospital. These hospitals were selected because they are the main referral hospitals with the largest NICU and high patient admission rates in the northern region of Jordan.

### 2.3. Population and Sample

The target population was NICU nurses in Jordan. The study sample involved NICU nurses working in the selected hospitals at the time of data collection who met the following inclusion criteria: working full‐time in the NICU, being available at the time of data collection, capable of reading and understanding English, and willing to participate. Based on a survey power analysis calculation using G∗Power 3.1 software to determine the required sample size for correlation analysis, at least 83 participants need to be surveyed to achieve a confidence level of 95%. After applying the inclusion criteria, 85 of the 104 NICU nurses at the selected hospitals were deemed eligible and personally invited by the researchers to participate in the study.

### 2.4. Data Collection

#### 2.4.1. Variables and Instruments

The study instrument was initially designed by the researchers based on CDC and WHO infection prevention guidelines to assess neonatal nurses’ knowledge and to observe their practices regarding HAI prevention guidelines. The study instrument consisted of three parts to measure the study variables as follows:

1. Nurses’ personal and professional characteristics were documented in a self‐administered form. The variables included the following: age, marital status, total years of nursing experience, level of education, and receiving formal training on HAI prevention before the study.
2. Nurses’ knowledge regarding HAI prevention guidelines was assessed by using a composite self‐administered knowledge questionnaire of 44 items. The questionnaire covered six major domains of infection prevention: (a) general knowledge about infection prevention guidelines and standard precautions (8 items), HH (9 items), the use of PPE (7 items), injection and medication safety (6 items), environmental cleaning and waste disposal (8 items), and reuse of medical equipment (6 items).
 The items were measured based on three responses: true, I don’t know, and false. The correct response was awarded one point, while the incorrect one and “I don’t know” were allocated zero points. The total scores ranged from 0 to 44, with the higher scores indicating more knowledge. To simplify the analysis and make it more meaningful, the total scores were converted to out of 100. Then, three main levels of participants’ knowledge about HAI prevention guidelines were used as follows: total score < 50% = poor knowledge, 50%–79% = fair knowledge, and ≥ 80% = good knowledge. A comparable classification system has been used in previous studies for infection control practices in NICU [16, 17].
3. Nurses’ actual practices of HAI prevention guidelines were observed by the first researcher, and adherence to the guidelines was documented and scored using a structured observation checklist. The observation checklist consisted of 46 items, categorized into five domains of infection control practices: HH (9 items), the use of PPE (10 items), injection and medication safety (10 items), environmental cleaning and waste disposal (10 items), and reuse of medical equipment practices (7 items).
 Each item in the checklist had a four‐point rating scale ranging from 0 to 3: “0” indicated that the nurse did not take the required infection control practice at all, “1” indicated that the nurse carelessly performed the practice, “2” indicated that the nurse performed the practice carefully but not in order, and “3” meant the nurse performed the practice carefully and in order. The mean value for each item was used to represent the participants’ adherence to HAI pronation practice at the workplace. To simplify the analysis and make it more meaningful, the total scores were converted to out of 100. Participants’ actual practices about HAI prevention guidelines were classified as poor knowledge when the total observation score was < 50%, fair when the score was between 50% and 79%, and good when the total score was ≥ 80% [16, 17].

#### 2.4.2. Initial Assessment and Psychometrics of the Study Instrument

The face and initial content validity of the developed tools were assessed by three faculty members with extensive expertise in maternal and pediatric nursing research, practice, and education. The study procedures’ feasibility and applicability, and the instruments’ clarity were assessed through a pilot study, which involved collecting data from 10 nurses from both settings to judge the items. Internal consistency reliability was assessed for the total sample through Cronbach’s alpha coefficients. The values were acceptable and adequate [18]; Cronbach’s alpha was 0.72 for the knowledge questionnaire, while it was 0.92 for the observation checklist items.

#### 2.4.3. Data Collection Procedures

Data were collected between March and May 2023. Data collection was carried out by the principal investigator (PI) for the majority of the time. In some instances, a trained research assistant, who was a skilled pediatric nurse with a master’s degree in nursing, provided assistance in data collection. The process is carried out in the following two phases.

##### 2.4.3.1. The Observation Phase

It involved collecting data by observing participants’ actual practice of HAI prevention guidelines using the developed observational checklist. A covert nonparticipant observation was performed. The observations were single‐blinded, where the researcher directly observed the HAI prevention practices for each eligible nurse while they provided direct care to neonates. The observations were sampled at different times to cover all possible working hours: morning, evening, and night shifts. Every single observation was performed until all domains of the HAI prevention checklist were covered for each observed nurse; this usually required 45–60 min of observation for each nurse.

##### 2.4.3.2. The Questionnaire Phase

It involved the researcher providing the knowledge questionnaire regarding the HAI prevention guidelines to the study participants. On average, it took 15 to 20 min to complete the self‐report questionnaire. The participant nurses were allowed to return the completed questionnaires to the researcher at their convenience. The researcher also provided their contact numbers and was mostly available at the data collection site to answer questions or provide further clarifications and explanations if needed.

### 2.5. Data Analysis

Statistical analysis was performed using the Statistical Package of Social Sciences (SPSS) Version 26.0. Descriptive statistics, including frequency, percentages, mean, and standard deviations, were used according to the variables’ level of measurement. Spearman’s rank correlation was performed to test the relationship between study variables, and Logistic regression was used to identify the most significant predictors of knowledge and observed practice of HAI prevention guidelines. Statistical significance was placed at *p* value ≤ 0.05.

### 2.6. Ethical Considerations

The study was approved by the Institutional Review Boards of the Jordan University of Science and Technology (Ref. 18/157/2023) and the Ministry of Health (Ref. MOH/REC/108/2023). The expected benefit of the study for neonatal nursing care exceeds the possible harm to the research subjects involved in the study. Direct observations of nurses are much better suited for providing a realistic assessment of actual HAI prevention practices. However, one concern of this study was that the knowledge questionnaire would trigger a Hawthorne effect regarding the observation of actual practices of HAI prevention interventions. Therefore, a covert observation was performed before the questionnaire was distributed to interested nurses. Participation was entirely voluntary, and both verbal and written informed consent were obtained prior to data collection. The study’s aims, risks, benefits, and the estimated completion time were explained verbally and on the questionnaire cover page. Participants were informed of their rights per the IRB guidelines, including confidentiality, the right of self‐determination, and the ability to decline or withdraw at any time before submitting the questionnaire. All questionnaires were numerically coded and deidentified, ensuring anonymity of the analyzed data.

### 2.7. Patient and Public Involvement

Patients or the public were not involved in the design, conduct, reporting, or dissemination plans of this research.

## 3. Results

### 3.1. Nurses’ Personal and Professional Characteristics

Out of 104 nurses working in both NICUs, a total of 85 nurses participated in the study. Participants’ personal and professional characteristics are detailed in Table 1. All nurses in this study were female, which is typical for neonatal nurses in Jordan, and aged mainly between 31 and 40 years (*n* = 58, 68.3%). Most nurses (*n* = 70, 82.3%) had a bachelor’s degree in nursing and more than 5 years of nursing experience (*n* = 61, 71.8%). Additionally, most nurses reported attending previous formal training in infection prevention and control (*n* = 61, 71.8%).

**TABLE 1:** Personal and professional characteristics of the participants (*n* = 85).

Variable	Frequency (*n*)	Percent (%)
Age		
25–30 years	22	25.9
31–35 years	39	45.9
36–40 years	19	22.4
> 40 years	5	5.9
Marital status		
Single	7	8.2
Married	75	88.2
Divorced	2	2.4
Widow	1	1.2
Workplace		
Hospital 1	48	56.5
Hospital 2	37	43.5
Years of experience		
1–5 years	24	28.2
6–10 years	39	45.9
11–15 years	11	12.9
16–20 years	9	10.6
> 20 years	2	2.4
Level of education		
Associate degree	3	3.5
Bachelor’s degree	70	82.4
Master’s degree	12	14.1
Previous training		
Yes	61	71.8
No	24	28.2

### 3.2. Neonatal Nurses’ Knowledge About Observed Practices of HAI Prevention Guidelines

#### 3.2.1. Knowledge

The mean score of the neonatal nurses’ HAI prevention knowledge was 36.18 (out of 44), with scores ranging from 21 to 42. As the scores were classified, 65% (*n* = 55) of the participants demonstrated good knowledge about HAI prevention guidelines, while 34.1% (*n* = 29) had fair knowledge, and only 1.2% (*n* = 1) had poor knowledge of HAI prevention.

#### 3.2.2. Observed Practices

The mean of the total practice scores of neonatal nurses’ HAI prevention guidelines was 88.21 (SD = 5.679), with scores ranging from 52 to 123 (out of 138). As the scores were classified, most of the participants (71.8%, *n* = 61) demonstrated fair levels of practice per the HAI prevention guidelines. Among the participants, 15.3% (n = 13) demonstrated poor practice levels, while only 12.9% (n = 11) indicated good practice of HAI prevention guidelines.

#### 3.2.3. Knowledge and Observed Practices of HAI Prevention Guidelines by Domains

Table 2 shows a range of disparities among participants regarding their knowledge and practice of HAI prevention. The highest knowledge scores were observed for the reprocessing of reusable medical equipment, while the lowest were related to the use of PPE. In terms of practice, the highest performance was also observed in the reprocessing of reusable equipment, whereas the poorest practices were noted in HH, followed by PPE use. Of particular note, all nurses demonstrated fair to good knowledge of HH; however, the majority showed fair to poor performance in HH practices, revealing a substantial knowledge–practice gap in this critical area. A similar pattern was evident in injection safety, where theoretical knowledge was stronger than observed practice. By contrast, PPE presented the opposite trend: Practice performance was relatively better than knowledge, suggesting that good practice in this domain did not necessarily depend on high levels of theoretical knowledge.

**TABLE 2:** Participants’ levels of knowledge and observed practices of HAI prevention guidelines per HAI domains.

HAI domains	Level of knowledge F (%)			Observed practices F (%)		
	Good	Fair	Poor	Good	Fair	Poor
Hand hygiene	36 (42.4)	49 (57.6)	0 (0.0)	7 (8.2)	39 (45.9)	39 (45.9)
The use of personal protective equipment	0 (0.0)	32 (37.6)	53 (62.4)	10 (11.8)	42 (49.4)	33 (38.8)
Injection and medication safety	65 (76.5)	20 (23.5)	0 (0.0)	8 (9.4)	75 (88.2)	2 (2.4)
Environmental cleaning and waste disposal	67 (78.8)	18 (21.2)	0 (0.0)	22 (25.9)	61 (71.8)	2 (2.4)
Reprocessing of reusable medical equipment	75 (88.2)	8 (9.4)	2 (2.4)	46 (54.1)	38 (44.7)	1 (1.2)

*Note:* Total scores for knowledge and observed practices: Good: ≥ 80%, fair: 50%–79%, poor: < 50%.

Abbreviation: HAI, hospital‐acquired infection.

### 3.3. The Relationships Among Study Variables

The correlation coefficient between knowledge and practice scores was not statistically significant (*r*_*s*_ = 0.101, *p* = 0.358). The correlation coefficients for the relationships among the nurses’ background information with the total knowledge and practice levels are detailed in Table 3. As depicted in the table, there was a significant association between neonatal knowledge levels and workplace setting (*p* = 0.02) and previous formal training (*p* = 0.001). Other background information was not significantly correlated with the nurses’ knowledge levels. Good practices of HAI prevention guidelines were positively correlated with level of education (*p* = 0.044), and negatively correlated with nurses’ age (*p* = 0.013), experience (*p* = 0.023), and the workplace setting (*p* = 0.001).

**TABLE 3:** Associated sociodemographic variables with knowledge and observed practices (*n* = 85).

Variables	Knowledge *r*_*s*_ *(p)*	Practice *r*_*s*_ *(p)*
Age	0.019 (0.40)	−0.267 (0.013)^∗^
Years of experience	0.014 (0.89)	−0.247 (0.023)^∗^
Educational level	0.154 (0.15)	0.219 (0.044)^∗^
Marital status	0.103 (0.34)	0.071 (0.50)
Workplace	−0.26 (0.02)^∗^	−0.510 (0.001)^∗∗^
Previous training	0.49 (0.001)^∗∗^	0.080 (0.41)

*Note:* r^s^ = Spearman’s correlation coefficient.

^∗^Statistical significance at *p* < 0.05 (2‐tailed).

^∗∗^Statistical significance at *p* ≤ 0.001 (2‐tailed).

Multivariable logistic regression results indicated that the length of experience and attending previous course training on HAI prevention were found to have significant predictive effects on nurses’ knowledge of HAI prevention guidelines (OR = 12.114 at *p* = 0.043 and OR = 4.21 at *p* = 0.001, respectively). However, the workplace has a significant independent predictive effect on nurses’ practice of these guidelines (OR = 0.79, *p* = 0.023). Other variables were found not to be significant predictors of knowledge or practice of HAI prevention guidelines among neonatal nurses.

## 4. Discussion

This study assessed neonatal nurses’ knowledge and observed practices of HAI prevention guidelines in two large teaching hospitals in Jordan. Preventing and managing infections are core healthcare practices, especially among nurses who are in ongoing and direct contact with the critically ill and vulnerable neonates in the NICUs. Nurses should be highly vigilant and are expected to be knowledgeable and highly trained in infection prevention to safeguard this vulnerable population. However, our findings showed that the neonatal nurses’ knowledge, practices, and training of HAI are far from this ideal state.

Overall, approximately two‐thirds of nurses demonstrated adequate knowledge, a result consistent with studies from other countries [19–21], as well as with findings among non‐NICU nurses in Jordan [22, 23]. This state of limited knowledge among nurses is only partially adequate to achieve safe healthcare and best practice of HAI prevention. In fact, all healthcare workers, especially nurses, should demonstrate at least above‐moderate, not to say excellent, levels of knowledge and awareness of HAI prevention guidelines. Without such knowledge, the provision of adequate and informed decisions concerning HAI prevention in practice would be questionable.

Variation across knowledge domains was also evident, a pattern reported in other studies [21, 24]. Notably, knowledge was particularly deficient in HH and injection and medication practices. These findings are concerning, given the central role of these practices in preventing HAIs, and highlight the need for regular, standardized in‐service training on HAI prevention that aligns with updated EB guidelines, with special emphasis on the areas where knowledge gaps are most evident.

This study also found no significant overall relationship between nurses’ knowledge and their observed practices of HAI prevention guidelines, emphasizing the complexity of translating theoretical knowledge into consistent practice. While knowledge levels were generally higher than practice across most domains, this did not predict greater adherence to infection prevention measures. These findings are consistent with previous research [26, 33], although other studies have reported a significant positive correlation between knowledge and practice [20]. The present aggregate results likely reflect domain‐specific discrepancies observed in this study; for instance, in areas such as HH and injection safety, theoretical knowledge exceeds practical performance, whereas for PPE, practice scores were higher despite lower knowledge. Such discrepancies point to a critical knowledge–practice gap, indicating that while knowledge forms an essential foundation, it does not always translate into consistent adherence to infection prevention measures, and suggesting that barriers beyond theoretical understanding play a critical role in shaping practice regarding HAI prevention in neonates, potentially across countries.

These barriers could arise from a combination of behavioral, organizational, and contextual factors, including workload and time constraints faced by nurses [20, 23], staff shortages [20], and limited HAI prevention resources and supplies, such as soap and sanitizers [20, 23, 25–27]. Addressing these barriers requires multifaceted strategies and interventions that integrate education, ongoing monitoring, and supportive workplace structures, along with the adequate and consistent provision of infection control resources in NICUs. Importantly, several of these barriers were also noted during field observations in this study, even though they were not directly assessed.

Attending in‐service training and the place of work were positively associated with nurses’ knowledge of HAI prevention, consistent with prior studies [22, 28, 29]. Training helps refresh nurses’ knowledge and connect them with the latest evidence‐based practices and guidelines. Furthermore, the length of experience was a key factor for knowledge among NICU nurses, which could be attributed to the fact that the longer the nurses spend in nursing care services, the more opportunities and time available to participate in in‐service HAI prevention training and update their knowledge. However, in line with our findings, training alone was not significantly associated with improved practice, aligning with [29]. Again, this suggests that while theoretical knowledge is necessary, it is insufficient to ensure adherence to prevention guidelines. What is required, therefore, beyond training, are complementary initiatives, such as ongoing monitoring and feedback, role modeling, embedding infection prevention practices into daily routines, and system‐level support, to bridge the gap between knowledge and consistent application of prevention practices.

Interestingly, negative correlations were found between nurses’ age, years of experience, and workplace with their practice of the HAI prevention guideline. This suggests that older or more experienced nurses were less adherent to recommended practices compared with their younger or less experienced colleagues, who are viewed as being more able to acquire, retain, and apply new knowledge to update their practices. These findings are in agreement with those of [30], who revealed statistically significant negative correlations between practices of HAI of the studied sample and their age and years of experience. Habituation, resistance to change, competing priorities, and reduced responsiveness to institutional monitoring have been shown to hinder compliance with HH protocols among experienced nurses. This suggests that while professional maturity may enhance theoretical understanding, it does not necessarily ensure the consistent application of infection prevention measures, and this is reflected in previous research as well [20].

Continuous professional development programs that emphasize behavioral change, reinforce adherence, and promote accountability across all levels of experience may help bridge this gap. Targeted interventions, such as mentorship programs, peer audits, and regular feedback mechanisms, may help experienced staff sustain adherence while also leveraging their expertise in NICU care [31, 32].

The workplace was identified as the strongest predictor of NICU nurses’ adherence to HAI prevention practices. Nurses working in the governmental hospital faced staffing shortages, limited resources, high nurse‐to‐patient ratios, and high admission rates of acute cases. This may reflect practice fatigue, whereby sustained workload pressure leads to shortcuts that compromise infection control. In addition, these constraints often require rapid responses in emergency situations, limiting the ability to fully adhere to and apply infection prevention guidelines. Comparable findings have been reported by [31, 32], who reported that heavy workloads and overcrowding impeded some infection prevention practices during critical situations, often balancing immediate patient needs against strict adherence. A Jordanian study [23] also found that nurses in private hospitals demonstrated higher knowledge and more positive attitudes toward infection prevention compared with those in governmental or teaching hospitals, likely due to differences in workload and institutional support. These findings emphasize that, while individual knowledge and motivation are essential, organizational and environmental factors play a decisive role in shaping infection prevention practices. Administrative strategies such as recruiting additional qualified staff, ensuring adequate resources, and supporting nurses in high‐acuity conditions are critical to improving overall adherence to HAI prevention guidelines in NICUs.

### 4.1. Strengths and Limitations

This study sheds light on a gap regarding NICU nurses’ knowledge and practice of HAI prevention—evidence that was limited in Jordan before this study. Another strength is its direct observation of actual practice, and this better reflects the reality than self‐reported research methods, which may be affected by socially desirable answers [18]. However, this study was limited in scope; hence, the results may not be generalizable beyond. Repeating it in diverse neonatal NICUs may yield different results. Although the researcher took measures to minimize a potential Hawthorne effect during the observation of actual practices, both Hawthorne and social desirability effects remain potential sources of bias. In addition, each nurse was observed only once, which may not fully reflect variations in practice across shifts, patient acuity levels, or over time. Together, these limitations suggest that future research should incorporate repeated observations across different contexts and triangulate findings with additional data sources to enhance validity and reliability.

## 5. Conclusion

The findings of this study indicate that, despite generally adequate knowledge, neonatal nurses exhibited unsatisfactory practices in applying HAI prevention guidelines within NICUs. These findings suggest a need for reinforcing infection prevention measures through continuous education, institutional support, and regular systemic monitoring. Incorporating more comprehensive infection prevention content into nursing curricula, alongside ongoing professional development, may help bridge the knowledge–practice gap. Future studies using larger, multicenter samples and mixed‐method approaches are warranted to further explore contextual factors influencing nurses’ adherence and to develop tailored interventions that strengthen infection prevention in neonatal care.

## Author Contributions

Conceptualization: Abedallah Y. Kasem, Huda Kh. Ahmad; design and methodology: Abedallah Y. Kasem, Huda Kh. Ahmad; data curation and analysis: Abedallah Y. Kasem, Huda Kh. Ahmad, Nadin M. Abdel Razeq, Alaa O. Oteir, WYGh; funding acquisition and supervision: Abedallah Y. Kasem; writing–original draft: Abedallah Y. Kasem, Huda Kh. Ahmad, Eihab A. Khasawneh, WYGh; validation and writing–review and editing: Abedallah Y. Kasem, Huda Kh. Ahmad, Nadin M. Abdel Razeq, Alaa O. Oteir, Eihab A. Khasawneh, Wafaa Y. AlGhazawi.

## Funding

The authors declare with gratitude that this work was supported by the Jordan University of Science and Technology under Grant No. 20230189.

## Conflicts of Interest

The authors declare no conflicts of interest.

## Data Availability

The data that support the findings of this study are not publicly available due to their containing information that could compromise the privacy of research participants and breach the institutional agreement but are available from the corresponding author upon reasonable request.
